# Providing Self-Led Mental Health Support Through an Artificial Intelligence–Powered Chat Bot (Leora) to Meet the Demand of Mental Health Care

**DOI:** 10.2196/46448

**Published:** 2023-06-19

**Authors:** Emma L van der Schyff, Brad Ridout, Krestina L Amon, Rowena Forsyth, Andrew J Campbell

**Affiliations:** 1 Cyberpsychology Research Group Biomedical Informatics and Digital Health Theme, School of Medical Sciences The University of Sydney Sydney Australia

**Keywords:** mental health, chatbots, conversational agents, anxiety, depression, AI, support, web-based service, web-based, deployment, stigma, users, symptoms, mental health care, self-led

## Abstract

Digital mental health services are becoming increasingly valuable for addressing the global public health burden of mental ill-health. There is significant demand for scalable and effective web-based mental health services. Artificial intelligence (AI) has the potential to improve mental health through the deployment of chatbots. These chatbots can provide round-the-clock support and triage individuals who are reluctant to access traditional health care due to stigma. The aim of this viewpoint paper is to consider the feasibility of AI-powered platforms to support mental well-being. The Leora model is considered a model with the potential to provide mental health support. Leora is a conversational agent that uses AI to engage in conversations with users about their mental health and provide support for minimal-to-mild symptoms of anxiety and depression. The tool is designed to be accessible, personalized, and discreet, offering strategies for promoting well-being and acting as a web-based self-care coach. Across all AI-powered mental health services, there are several challenges in the ethical development and deployment of AI in mental health treatment, including trust and transparency, bias and health inequity, and the potential for negative consequences. To ensure the effective and ethical use of AI in mental health care, researchers must carefully consider these challenges and engage with key stakeholders to provide high-quality mental health support. Validation of the Leora platform through rigorous user testing will be the next step in ensuring the model is effective.

## Digital Mental Health

Mental health is a significant public health concern. Globally, it is estimated that 1 in every 8 people lives with a mental disorder [1]. Mental health service use for depression was estimated to range from 33% in high-income countries to 8% in low- and middle-income countries [2]. A significant gap exists between mental ill-health and access to clinical services. Further, global estimates have not yet captured the impact of the COVID-19 pandemic. Some preliminary estimates suggest that an additional 76.2 million cases of anxiety disorders could be attributed to COVID-19 [3].

The outbreak of COVID-19 and subsequent stay-at-home requirements from governments across the world were the precursors to a significant increase in the demand for mental health services [4]. Amid uncertainty in health and employment, individuals reported that mental ill-health was exacerbated [4]. In response, the World Health Organization stated, “the scaling-up and reorganization of mental health services that is now needed on a global scale is an opportunity to build a mental health system that is fit for the future” [5].

Web-based mental health services are a key to improved service delivery that can be augmented to meet the needs of the changing times ahead [6]. Telehealth appointments, whose reach was expanded during the pandemic, are an effective way digital health was deployed. Research suggests that the expansion of telehealth services was generally well-accepted [7]. However, long wait times limit the accessibility of telehealth services. Moreover, the demand for clinically effective web-based mental health tools and services far exceeds the supply [8,9]. COVID-19 required a significant rethinking and restructuring of what effective mental health care service delivery looks like. With this, evidence-based, efficacious, and easily scalable digital mental health services are increasingly needed.

## Artificial Intelligence in the Context of Mental Health Treatment

Artificial intelligence (AI) is the ability of computer systems to display intelligent behavior by analyzing an environment and taking action with some degree of autonomy [10]. In health care, AI is prominently used in the context of predicting, detecting, and treating illness. This is particularly useful when developing solutions for large-scale problems. Burgeoning examples of this can be found in clinical settings [11] as well as in translational medical research [12,13]. Another way AI is being used is in chatbots, where it has the potential to make an impact in the area of mental health [14,15]. Chatbots are software that engages in dialog with individuals using natural language [16].

The advent of these mental health bots has the potential to be useful in several ways. For one, therapeutic chatbots can provide a platform for individuals to engage at any hour of the day in self-help. Ready access to digital therapies and tools is important given that distressed individuals may seek help outside business hours. Bots also have the potential to triage individuals who feel stigmatized by the current health care model and who otherwise would not be comfortable accessing treatment. Reluctance to access help due to the fear of being judged or labeled is a significant barrier for individuals to access mental health support [17]. Men and some minority groups report stigma as being particularly prolific in this context [18,19].

Woebot [20] and Wysa [21] are 2 examples of AI chatbots in the field of mental health. Woebot uses cognitive behavioral therapy and was found to significantly reduce symptoms of depression and anxiety in young adults [15]. Wysa also reported a reduction in self-reported symptoms of depression and reported that 68% of the cohort found the app helpful and encouraging [14]. These tools show potential for AI mental health chatbots; however, critiques have been made about the size of the sample in the validation studies and the lack of research on their long-term efficacy [22]. Given the potential for AI-powered tools to address mental ill-health at scale, further research into the development and validity of this technology needs to be completed.

## Challenges of AI in Mental Health Treatment

Providing mental health treatments necessitates ethical scrutiny, especially when incorporating AI into brief assessments and the support of users. The difficulty of designing ethically robust interventions with AI is that ethical design and review cannot remain a one-off, as the technology itself is continuously developing for both the user and clinical providers [23]. Transparency in the development and limitations of AI are important considerations. For example, how an algorithm works to predict and learn is especially important to understand in the context of mental health, particularly regarding how it relates to patient safety and the potential provision of clinical advice. Further, confidentiality and anonymity are a particular concern for mental health tool users, given the stigma associated with mental ill-health [24,25], as are data ownership and privacy rights, which are difficult but important elements to manage [26].

## The Proposed Model

In consideration of the aforementioned challenges of AI development for mental health support, the AI platform Leora has incorporated a model of development that is transparent, ethical, and user-focused with regard to data privacy. An understanding that AI is currently best suited as a mental health support tool and early service entrance assistant for users has guided the development of Leora toward its focus as an AI mental health support program.

Leora is a conversational agent that leverages AI to engage in discussions with users about their mental health. Accessible via a web browser and mobile app, this digital toolkit has the capacity to assist individuals with mental ill-health by assessing, monitoring, and managing minimal-to-mild symptoms of anxiety and depression. Further, Leora provides evidence-based strategies to cope with distress and promote well-being. Leora is positioned as a web-based self-care coach and offers easily accessible, personalized, and discreet mental health support.

## Leora’s Roadmap for AI Support

**Figure 1:**
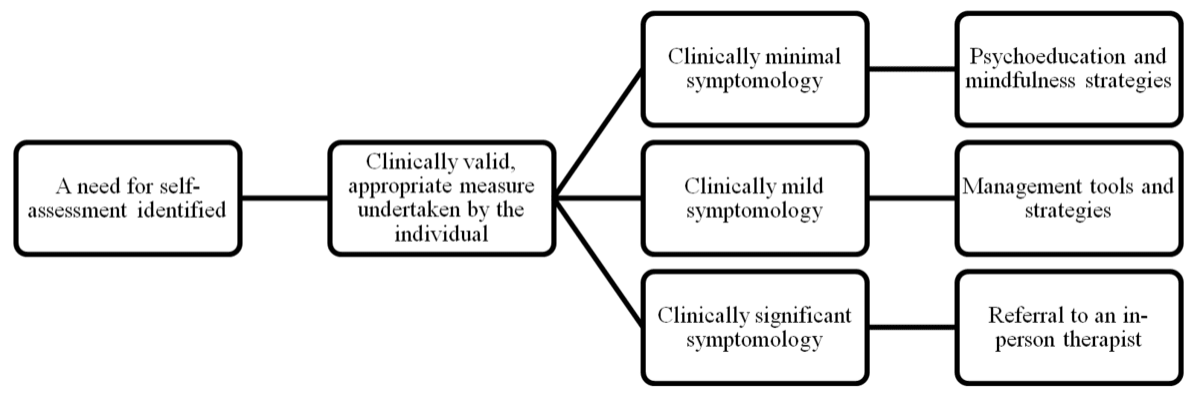
Road map for self-assessment of mental ill-health on the Leora platform.

An interaction with Leora is illustrated in Figure 1. The first step is identifying the individual’s needs. This is an important component of patient-centered care and is associated with more promising patient outcomes [27,28]. AI can gauge the appropriate clinical need via a standardized evaluation tool for anxiety and depression, which are the most common mental health concerns [1]. It can do this by using natural language processing—an algorithm that allows a computer to understand language as it is spoken and written [29].

An important ethical consideration in developing and implementing AI digital health solutions in the mental health field is that solutions and services must provide clinically appropriate recommendations [30]. By integrating the standardized measures of the Generalized Anxiety Disorder 7-item scale (GAD-7) and the Patient Health Questionnaire 9-item scale (PHQ-9) into the platform, recommendations and referrals will be clinically valid in terms of criterion, construct, factorial, and procedural validity (see [31,32] for GAD-7 validity testing and [33,34] for PHQ-9 validity testing). Individuals with clinically significant scores on these measures will be directed to mental health services to book an in-person therapy session. Individuals with low or no indication of anxiety and depression symptoms will be directed to psychoeducation and mindfulness strategies, deemed efficacious for those needing to understand their mood and cognitive changes. This approach to care is standardized and is well-used by many web-based mental health services [35,36]. Individuals with mild depression and anxiety scores will be directed to strategies to manage these factors based on mental health first aid techniques grounded in brief cognitive behavioral therapy [37,38] and Acceptance and Commitment Therapy, also known as mindfulness [39].

**Figure 2:**
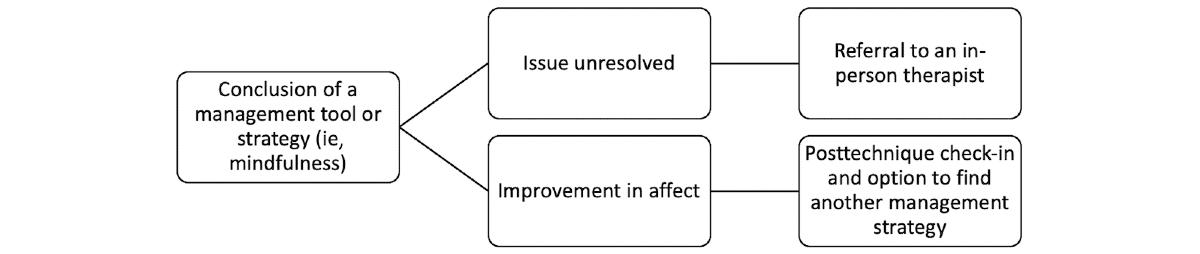
Assessing the effectiveness of the mental health support strategies offered by Leora.

Figure 2 illustrates a flowchart of the evaluation of mental health support strategies that Leora would offer an individual. If strategies to reduce the impact of depression and anxiety were not considered helpful by the individual, the platform would refer the user to another strategy that may provide improved support. If the strategy is considered effective by the individual, posttechnique support and maintenance sessions may be offered.

## Blending AI and Humanistic Therapy

**Figure 3:**
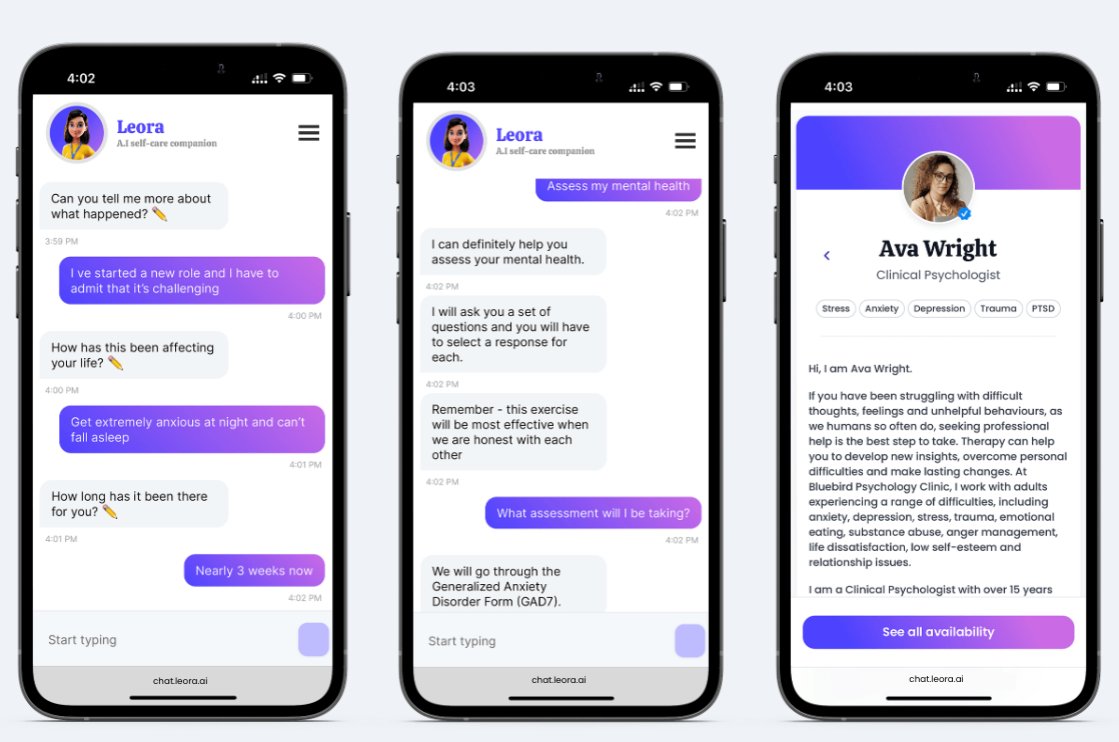
Leora platform interface: chatting to the conversational agent, self-assessment, and booking therapy.

The platform blends AI with authentic, human-first, psychological support known as humanistic therapy [40]. The conversational agent can engage in meaningful conversations with the individual, conduct standardized assessments, and book therapy for those who need more support (see Figure 3 for the interface examples).

Patient safety is paramount throughout client engagement. Leora has developed “escalation pathways” into the design of triaging patients. A self-assessment that indicates an individual has clinically significant levels of anxiety and depression will be referred to appropriate services, such as a psychologist or a local mental health emergency contact (eg, a mental health crisis website or phone service). In the instance that the client meets the criteria for a referral process, the platform would assist the user to access an appointment with nonconfrontational wording, for example, “You might want to get support beyond what I can help you and that’s not a problem at all! I know a network of experienced mental health professionals. I can assist you in booking sessions with them.”

## Evaluating the Efficacy of Care

Accessibility, equity, clinical effectiveness, user acceptability, and satisfaction, as well as service efficiency, are components of ethical digital health solutions [41] and can be evaluated by data drawn from Leora. As such, data collected from the cycle of support and evaluation of individual users can be assessed and integrated back into the model to improve the AI service. This iterative design process, characteristic of AI and machine learning models, gives Leora scope to improve by integrating the experiences of a variety of users. Independently, qualitative feedback and internal or external user experience testing are additional layers of evidence to cross-reference with the review data of client sessions with Leora.

## Data Safety, Encryption, and Anonymity

Leora is stored on Amazon Web Services (AWS) cloud servers and is secured by virtual private cloud networking configurations. Data are protected from exposure to the public-facing internet through the strict configuration of security groups, subnet settings, and the use of identity access management. This establishes the highest degree of privacy for collected data and ensures that it cannot be leveraged for commercial use. Leora’s architecture is protected by the AWS Encryption Software Development Kit (Amazon Web Services, Inc), which uses envelope encryption. This form of multilayered encryption involves first generating a key for the data chunk at the application layer and then wrapping or encrypting this key again at the storage layer, creating a hierarchy of keys with access governance controlled by identity access management [42]. To decrypt the encrypted message, the AWS Encryption Software Development Kit uses the wrapping key to decrypt at least one encrypted data key. It can then decrypt the ciphertext and return a plaintext message. Leora is compliant with the Australian Privacy Policy [43] and gives users of the platform the option of gaining access (through authorization and verification tokens) and deleting their data. While one of the functions of Leora is to build connectivity between users and therapists, it also puts the choice of what data is shared in the hands of users.

## Transparency of Scope

Appropriate acknowledgment of the scope of support in digital mental health solutions is a pillar of ethical development and patient care [30]. It is critical for conversational agents to be transparent about the scope of their abilities. Leora is not designed to assist with mental health crises. Clear warnings and postings of this message appropriately indicate to users the scope of the platform. Abuse, self-harm, severe mental health conditions that may cause feelings of suicide, and any other medical emergencies are generally out of the scope of conversational agents. Leora cannot and will not offer medical or clinical advice to individuals, so if presented with a mental health crisis it will instead direct clients to emergency mental health services.

## Discussion

### Principal Findings

As the use of AI-powered mental health chatbots becomes more widespread, ethical design and development must be considered. One pillar of the ethical integration of AI into mental health treatment is trust and transparency. Clinicians and patients may be hesitant to trust these platforms if they do not have a basic understanding of how the algorithms work to provide care [44]. To address this issue, it is imperative that the detailed development, testing, and deployment of AI chatbots be transparent to clinicians and consumers. This means clearly explaining the algorithms and processes that the chatbots use to generate responses, as well as the limitations of the technology and any and all risks to users. By providing this information, clinicians and patients may make more informed decisions about the advice provided by an AI chatbot and how to use it effectively.

Another important consideration for conversational agents is the potential for bias and health inequity. This is especially pertinent for groups with low health literacy levels, who may not be native speakers or people living with a disability. To ensure that chatbots are accessible, developers must design the technology to be user-friendly and easy to understand. This may involve providing clear and concise explanations of the chatbots’ responses as well as offering multiple ways for users to interact with the technology (eg, through text, voice, or other means).

McGreevey et al [30] suggest that there are several research and developmental considerations in the development of effective and ethical AI conversational agents. Some of the key research and development challenges for researchers going forward include evaluating the effectiveness of different approaches or tones for conversational agents, such as empathetic and stoic tones, terse and engaging delivery, and gendered delivery of care. Understanding the reasons why some patients stop using conversational agents is also crucial for improving technology. Longitudinal evaluation of patient outcomes with conversational agents is also crucial. This can help researchers determine the effectiveness of the technology in delivering mental health care across demographics and differing client needs. By addressing these research and development questions, researchers can help ensure that AI-powered conversational agents are used effectively in the introductory phase of this technology and provide the greatest benefit to individuals in the long term.

Further research should also consider the changing needs of patients. Ratheesh and Alvarez-Jimenez [6] note that with improvements in technology and an overall improved comfort with technology, it is possible for technology to change and adapt to the expectations and needs of its users. Given that research suggests that the relationship between the individual and clinician is one of the strongest correlates of successful treatment [45], further research is required to determine patient values and preferences for the role of AI-powered mental health support, such as in providing a pathway to developing successful therapeutic alliances.

Supporting innovation in the development of AI-powered conversational agents for mental health is essential to ensuring that patients have access to the most advanced and effective treatments available. However, it is also important to balance this goal with the need to protect patients. While the ethical development of AI is paramount, conservative approaches to the implementation of cutting-edge technology need to be balanced with supporting innovation with the potential to positively impact individuals on a large scale. One way to support innovation while still protecting patients is to carefully consider the potential risks and benefits of any new technology before it is introduced. This might involve conducting clinical trials to evaluate the safety and effectiveness of the technology, as well as engaging with key stakeholders such as patients, health care providers, and ethics experts to ensure that any potential concerns are addressed. Ultimately, supporting innovation in the development of AI-powered conversational agents for mental health will require a careful balance between boundary-pushing and the need to protect patients.

Moving forward, it is imperative for AI-powered mental health support to test the clinical outcomes for specific mental health disorders. While previous research has tested usefulness, acceptability, and perception [46], clinical effectiveness and reduction in the symptomology of depression, distress, and stress need to be tested more rigorously. A recent meta-analysis concluded that while there was promise for conversational agents, the evidence for them was currently weak due to the limited number of clinical trials and the high estimated risk of bias in the current evidence base [47]. Future research should aim to rectify this problem using a combination of participant-centered action research and traditional randomized control trials of AI-powered chatbots with a comparative therapeutic approach (eg, human-led solution-focused counseling).

### Conclusions

Digital mental health development is an important and necessary component of mental health service delivery. The Leora platform is a promising addition to self-led mental health help that leverages AI and humanistic therapy to support and triage individuals to appropriate mental health services. This is significant given the increasing demand and limited supply of mental health treatments worldwide. Notably, it meets the agreed pathways and development standards set by the Australian Government’s National Digital Mental Health Framework [48]. The integration of AI-powered chatbots into mental health service delivery is a logical innovation toward increasing equitable and free mental health service access globally. It necessitates certain ethical considerations to foster trust and transparency between client users and service providers. It is therefore essential in these early years of AI mental health support innovation that the development and deployment of AI chatbots are transparent to clinicians and consumers in order to rapidly and safely meet client demand and provide for best-practice use of the technology as an adjunct to existing clinical services. To do this, a layperson’s explanation of both the algorithms and processes that leverage chatbots’ data and the limitations of the technology will ensure a robust model of care. Ideally, this technology should aid clinicians and patients in making informed decisions about mental health treatment and services. Developers should remain vigilant against clinical and user experience bias. Moreover, equity should remain at the forefront of designing these tools to promote user acceptability, particularly for groups with low health literacy, nonnative speakers, or those living with a disability.

As AI for mental health support refines over the coming years, specifically for triaging clients in mental distress and conducting accurate psychometric testing, it has the potential to significantly benefit the strained traditional mental health system (ie, general practitioners, psychologists, and psychiatrists). By incorporating sophisticated yet clinician-monitored data sharing between AI systems like Leora and digital patient health records, this technology has the ability to provide the results of clinically validated longitudinal measures to these health care professionals, which in turn may expedite the treatment and recovery process of individuals with complex cases in a timely manner. The next step will be to test the Leora platform’s ability to deliver timely and effective mental health treatment.

## Abbreviations

AI: artificial intelligence
AWS: Amazon Web Services
GAD: Generalized Anxiety Disorder
PHQ: Patient Health Questionnaire
